# Global Dialysis Perspective: Maldives

**DOI:** 10.34067/KID.0000000000000261

**Published:** 2023-10-19

**Authors:** Ahmed Abdulla, Ibrahim Shiham, M. Razeen Davids

**Affiliations:** 1 Department of Nephrology, National Uro Renal and Fertility Centre, Indira Gandhi Memorial Hospital, Malé, Maldives; 2 Division of Nephrology, Faculty of Medicine and Health Sciences, Stellenbosch University and Tygerberg Hospital, Cape Town, South Africa

**Keywords:** chronic dialysis, CKD

## Introduction

The Republic of Maldives consists of 1210 islands spread across the equator in the Indian Ocean, south-southwest of India. It has 187 inhabited islands that are divided into 20 administrative atolls. The total population is 557,426 (2020 data), with a local population of just 379,270 people.^1^ The elderly population (65 years and older) comprised 5% of the population in 2022.^2^ Maldivians are an Indo-Aryan ethnic group who share the same culture and speak the same language, Dhivehi. English is widely spoken across the country.

The economy is dominated by the tourism industry. Malé is the capital and home to 38.7% of the population. The greater Malé area, consisting of the capital Malé, Hulhumalé, and Villimalé, has half of the doctors in the Maldives and most hospital beds.^1^

It is an upper middle-income country with a gross domestic product of USD 3.32 billion in 2020 and spends approximately 10% on the health sector.^3^ The state provides unlimited universal health insurance for all Maldivians; this cost USD 130 million in 2020^1^

**Figure 1:**
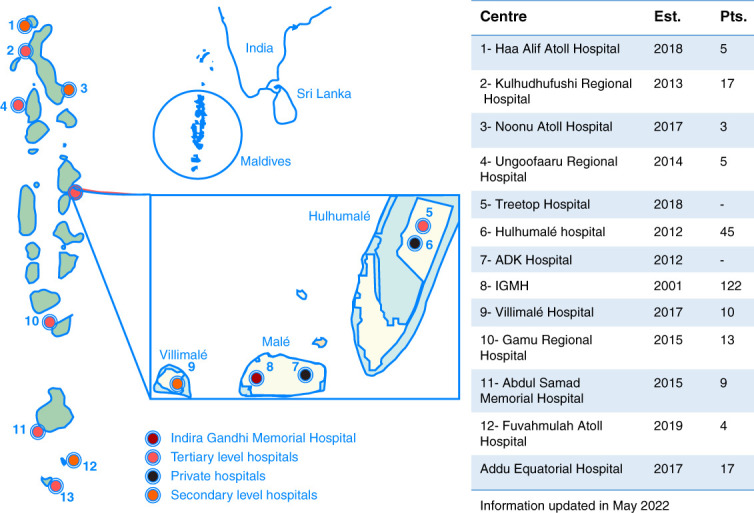
The atolls of Maldives with the greater Malé area in the inset, showing the distribution of dialysis centers across the country.

The health sector consists of a four-tier system. In the capital, there is a tertiary hospital, Indira Gandhi Memorial Hospital (IGMH), and there are five regional tertiary hospitals (Figure 1) which receive referrals from secondary-level atoll hospitals.

## Epidemiology

There is a paucity of data on CKD and RRT in the Maldives, and we therefore sent out questionnaires to all dialysis centers in May 2022. There was a 100% return from the public hospitals and a partial response from private hospitals. Cross-sectional data collected in 2019 at IGMH were used for characteristics of dialysis patients.

Maldives Health Data 2020 reports the number of deaths due to nephritis or nephrosis at 3% of noncommunicable disease deaths.^4^ According to the World Health Organization Global Health Observatory, kidney diseases in Maldives are the fourth most common cause of death (15.1 per 100,000).^5^

Among patients undergoing dialysis in IGMH, hypertension was the most commonly reported cause of kidney failure (Table 1). Most patients are hypertensive at the time of presentation, and there is a tendency to attribute hypertension as the cause of the kidney failure. This is likely overdiagnosed because some of these patients may have diabetic nephropathy which is not detected when patients present late, and others may have underlying glomerulonephritis or other unknown causes. This issue has also been highlighted by the South African Renal Registry.^6^

**Table 1:** Characteristics of patients undergoing dialysis at Indira Gandhi Memorial Hospital (data from 2019, *N*=102)

Characteristic	Value
Age, yr, median (IQR)	60 (47–67)
Male, %	46
**Primary kidney disease diagnosis, %**	
Hypertension	47.1
Glomerulonephritis	19.6
Diabetes mellitus	24.0
Cystic disease	1.9
Unknown	9.8
**Dialysis initiation, %**	
Urgent first hemodialysis	48.5
AVF at initiation	41.7
Thrice-weekly hemodialysis	80
Hemoglobin, g/dl, mean±SD	10.9±2.0
Albumin (g/dl), mean±SD	3.9±0.5

IQR, interquartile range; AVF, arteriovenous fistula.

Data from government-run centers indicate that there are 250 patients on hemodialysis and none on peritoneal dialysis (PD). There were an additional 17 patients in private hospitals, yielding a total of 267 patients.

Although most patients settle on an island with a dialysis center, 13 (5.2%) were traveling long distances by sea to reach a dialysis center and return on the same day. Patients use a public sea ferry service or hire speedboats, which take 30 minutes to 2 hours to reach dialysis centers located 10–45 km away.

## Dialysis Services

Hemodialysis was introduced in the Maldives at IGMH in June 2001 with a dialysis unit donated by a private company, Universal Enterprises. By 2014, the unit had expanded from having four dialysis machines to a facility with 12 machines. In November 2014, a brand-new center, Dr Ahmed Razee Memorial Dialysis Center, with 20 machines, was opened under the newly established division of nephrology. Nephrology services in IGMH were expanded to offer continuous RRT in mid-2014. The first acute PD in the country was performed in late 2014 on an infant with multiorgan failure.

Patients on hemodialysis at IGMH have access to a weekly walk-in hemodialysis clinic, and hemodialysis nurses can refer patients to this clinic. There are no regular consultation services by nephrologists for patients who are treated outside IGMH. These patients consult nephrologists through routine nephrology outpatient clinics, online clinics, or private consultations.

RRT is fully covered by insurance, irrespective of patients' prospects of having a kidney transplant. The economic effect on families resettling on a different island is significant because of the loss of employment and the cost of accommodation.

There is no PD program running in the country. PD is not covered under the national insurance scheme, and this is a major obstacle to its establishment. With no established continuous ambulatory PD program and no government funding, there is an inherent bias against PD among patients and health care professionals who are unfamiliar with the modality. There is also a lack of support at the government policy level, with current efforts by government focusing on establishing hemodialysis centers across the country. There is reluctance from private suppliers to import PD solutions because of the losses incurred from unsuccessful previous attempts at establishing PD as a treatment modality in Maldives.

Water purification systems are available at each dialysis center, with regular testing for chemical purity and bacterial colony counts. There is a lack of availability of endotoxin testing, analysis for heavy metals, and the recommended culture media in public hospitals. There have been no reports of febrile episodes which might suggest poor water quality despite the lack of availability of recommended bacterial culture media and endotoxin testing. One private hospital (Treetop Hospital) offers hemodiafiltration and does endotoxin testing. Current efforts are to facilitate these tests through existing state-run water testing authorities.

Table 2 summarizes the dialysis services in the Maldives. There are 11 public sector dialysis centers, with 65 dialysis machines (Figure 1). Centers provide dialysis 3–7 days per week, with two to four sessions daily, depending on patient numbers. There are low numbers of patients in peripheral centers and larger numbers in the greater Malé area, where dialysis is scheduled daily, except for Fridays. All patients have twice- or thrice-weekly treatment sessions of 4 hours each.

**Table 2:** Dialysis services in the Maldives (May 2022)

Variable	Value
Total no. of patients on hemodialysis (pmp)	267 (704 pmp)
No. of patients in the public sector	250
No. of patients in the private sector	17
No. of dialysis nurses in public sector	81
No. of nephrologists in private and public sectors	5 (8.9 pmp)
Nurse to patient ratio in dialysis units	IGMH 1:6 Others 1:1–1:3
Average length of dialysis sessions	4 h
Vascular access, %	AVF 86.4 AV graft 0 Tunneled catheter 13.6

pmp, per million population; IGMH, Indira Gandhi Memorial Hospital; AVF, arteriovenous fistula; AV, arteriovenous.

Of the 250 patients in the public sector, there are 38 patients on twice-weekly and 212 patients on thrice-weekly dialysis. There are 122 patients in the hospital where a nephrologist is available and 128 in the other hospitals. There are four machines dedicated for hepatitis B or C positive patients. Dialyzer reuse is not practiced. A dialysis machine dedicated for acute dialysis is available in four centers.

Acute dialysis is available in settings with resources for managing the critically ill. Patients are transferred by speedboats or chartered flights to tertiary centers, especially to IGMH.

Although efforts are made to prepare patients for dialysis with an arteriovenous fistula (AVF), approximately half the patients start dialysis on an urgent basis using a temporary catheter (data from the main referral hospital, IGMH, Table 1). AVF creation is performed by a local vascular surgeon and trained urologists. However, fistula salvage procedures for AVF thrombosis are seldom undertaken.

Patients have regular laboratory investigations performed at 1- and 3-month intervals and are encouraged to have regular consultations. The investigations, consultations, and medication are free of charge.

## Human Resources

Nephrology services started in 2001 when IGMH obtained the services of an Indian nephrologist, Dr. Lakshmi Kant Tripathi, who stayed for 2 years. There was then a hiatus of more than 10 years until the first local nephrologist joined the service in 2014.

At present, there are no nephrologists stationed in the public sector outside the greater Malé area. In 2022, Maldives had a nephrologist density per million population (pmp) of 8.9, higher than neighboring India which has 1.9 pmp^7^ and other upper middle-income countries such as Mauritius (7.5 pmp) and South Africa (2.5 pmp).^8,9^ The median number of nephrologists for upper middle-income countries was 10.8 pmp in 2019.^10^ However, this does not paint a true picture of the scenario in the Maldives because there is only one Maldivian nephrologist in the country.

There are no local training facilities for nephrologists, and candidates interested in nephrology as a career have limited access to training positions abroad.

The Maldives has no formally trained nephrology nurses. Nurses do 1–2-month rotations at the IGMH dialysis center to learn the practical aspects of dialysis. At present, there are 81 nurses in 11 public centers. There is a lack of opportunities for further training, and no formal nursing needs assessment has been performed. Dialysis centers in the periphery are managed by nurses and supported by physicians and general practitioners. Barriers to the training of nephrology nurses include a high turnover rate among nurses, lack of available training placements, lack of financial support, and, before May 2023, the lack of formal positions for specialized nurses.

## Vascular Access for Dialysis

There are 216 (86.4%) patients in the public sector using arteriovenous fistulae and 34 (13.6%) patients using tunneled catheters. There is no patient with an arteriovenous graft. Patients with CKD stage 5 and progressive worsening of kidney function are referred to a vascular surgeon for the creation of an AVF.

## Transplantation

All eligible patients are offered kidney transplantation. A patient who has a suitable donor can undergo a kidney transplant in India or Sri Lanka. According to the data obtained from National Social Protection Agency, the universal insurance scheme (Aasandha) has covered the cost of 70 transplants since 2018, each costing approximately USD 13800. The Aasandha scheme covers all medical expenses, including the cost of immunosuppressants. Transplantation is more cost effective than hemodialysis, which costs USD 3735 per patient per year in the state sector.

A committee, led by the National Uro Renal Center, is working toward a kidney transplant program in Maldives. This transplant committee has focused on developing infrastructure, human resources, training, and transplant legislation.

## Challenges and Solutions for Present and Future

Maldives is a country which consists of many islands, and a PD program would therefore be an ideal RRT modality. However, the future looks bleak for the establishment of a PD program, with government focusing instead on the establishment of hemodialysis centers; however, there are preliminary discussions being held with stakeholders regarding establishing a PD program.

A transplant program does not yet exist in the country. Efforts are ongoing for human resources, and infrastructure development and human organ donation legislation is in the pipeline.

The training of nurses and nephrologists remains challenging because of the lack of training opportunities and financial support. Maldivians are eligible for The International Society of Nephrology fellowships, and using this opportunity together with additional funding to cover the required 2-year training period will be the way forward.

There is progress on addressing the lack of reliable data on kidney diseases in Maldives. A CKD registry and a transplant registry are being developed, and the establishment of a national renal registry would be the next important step.
